# *Saccharomyces cerevisiae* surface display of endolysin LysKB317 for control of bacterial contamination in corn ethanol fermentations

**DOI:** 10.3389/fbioe.2023.1162720

**Published:** 2023-04-06

**Authors:** Shao-Yeh Lu, Siqing Liu, Maulik H. Patel, Kristina M. Glenzinski, Christopher D. Skory

**Affiliations:** ^1^ Renewable Product Technology Research Unit, National Center for Agricultural Utilization Research, USDA, Agricultural Research Service, Peoria, IL, United States; ^2^ Oak Ridge Institute for Science and Education (ORISE), Oak Ridge, TN, United States

**Keywords:** yeast surface expression, phage endolysin, LysKB317, peptidoglycan hydrolase, fuel ethanol, *Limosilactobacillus fermentum*, antimicrobial, *Saccharomyces cerevisiae*

## Abstract

Control of bacterial contamination in bioethanol fermentation facilities has traditionally relied on chemical-based products such as hop acids and use of antibiotics. Recent emphasis on antibiotic stewardship has prompted new research into the development of alternative approaches to microbial remediation strategies. We recently described a recombinant peptidoglycan hydrolase, endolysin LysKB317, which inhibited *Limosilactobacillus fermentum* strains in corn mash fermentation. Here, *Saccharomyces cerevisiae* EBY100 was used to anchor recombinant LysKB317 using cell surface display with the a-agglutinin proteins Aga1p–Aga2p. Immunostaining and confocal fluorescence were used for localization of the extracellular interface of the cells. Yeast surface-expressed endolysin demonstrated an 83.8% decrease in bacterial cell counts compared to a 9.5% decrease in control yeast. Recombinant *S. cerevisiae* expressing LysKB317 used for small-scale corn mash fermentation, when infected with *L. fermentum*, could proactively control bacterial infection for 72 h with at least 1-log fold reduction. Analysis of fermentation products showed improved ethanol concentrations from 3.4% to at least 5.9% compared to the infection-only control and reduced levels of lactic and acetic acid from 34.7 mM to 13.8 mM and 25.5 mM to 18.1 mM, respectively. In an optimized yeast surface display system, proactive treatment of bacterial contaminants by endolysin LysKB317 can improve fermentation efficiency in the presence of *L. fermentum* contamination.

## Introduction

Antibiotic and chemical treatments such as chlorine dioxide and hop acids have traditionally been used to mitigate bacterial contamination in commercial fuel ethanol fermentation facilities (Ruckle and Senn, 2006; Meneghin et al., 2008; Muthaiyan et al., 2011; Martin et al., 2015; Walter et al., 2019). Continuous yeast propagation and non-sterile fermentation conditions used in most commercial ethanol fermentation facilities frequently result in chronic and unpredictable acute bacterial infection that leads to stuck fermentations (Bischoff et al., 2009; Beckner et al., 2011). Costly facility shutdowns for cleaning caused by stuck fermentations are associated with acetic and lactic acid concentration buildup, byproducts of lactic acid bacteria (LAB), of which *Lactobacillus* and *Limosilactobacillus* species are the main contaminant that hinder yeast growth and ethanol production. (Skinner and Leathers, 2004; Skinner-Nemec et al., 2007). Antimicrobial resistance (AMR) is a significant global concern clinically in both humans and animals (Tang et al., 2017; Woodworth et al., 2018) and industrial and agricultural settings (Compart et al., 2013; Mann et al., 2021; Sorinolu et al., 2021). The recent shift toward reduction in non-medical usage and antibiotic stewardship has resulted in the development and utilization of antibiotic alternatives, such as antimicrobial peptides and enzybiotics (Branco et al., 2022). The drawback of scaling up the production and purification of antimicrobial peptides or enzybiotics products to treat large-scale fermentation facilities is the associated cost (Bommarius et al., 2010).

Bio-mitigation of bacterial infection using genetically engineered yeast such as *Saccharomyces cerevisiae* expressing recombinant proteins like the endolysin can be cost-effective and permit proactive treatment of microbial contamination. Endolysins (also known as lysins) are peptidoglycan hydrolase enzymes utilized by bacteriophages at the end of the lytic cycle to degrade the host bacterium cell wall from within (Fischetti, 2008; Schmelcher et al., 2012). Supplementing corn mash fermentations with purified endolysins (LysA and LysA2) in *S. cerevisiae* demonstrated that these endolysins can remain active and reduce contaminants such as *Limosilactobacillus fermentum* over a period of 72 h. While attempts to secrete these endolysins were unsuccessful, intracellular expression of LysA and LysA2 in *S. cerevisiae* still provided a reduction in *L. fermentum,* presumably from yeast cell autolysis (Khatibi et al., 2014). Subsequent attempts by others to secrete LysA2 were unsuccessful due to low secretion efficiency and low enzyme efficacy (Kim et al., 2018).

Use of a yeast surface display platform to anchor recombinant proteins to yeast cell wall has been widely adapted in the biomedical field for antibody and antimicrobial peptide engineering, protein stability, protein–protein interaction, and enzyme metabolic studies (Cherf and Cochran, 2015; Wen et al., 2018; Medina-Cucurella et al., 2019; Lau et al., 2021; Luo et al., 2021; Fiebig et al., 2022; Kuroda and Ueda, 2022; Raeeszadeh-Sarmazdeh and Boder, 2022; Teymennet-Ramirez et al., 2022). A common yeast display system involves fusing recombinant proteins to the a-agglutinin mating protein Aga2p and expressing extracellularly in yeast that secrete the ß 1,6 glucan-anchored Aga1p domain of a-agglutinin to form an Aga1p–Aga2p complex *via* two disulfide bonds (Wang et al., 2005; Cherf and Cochran, 2015). In this platform, epitopes can be detected with the protein of interest when correctly expressed, folded, and surface-displayed. In addition, protein aggregation in the cytoplasm is reduced, and the need for protein purification in the downstream process is eliminated (Teymennet-Ramirez et al., 2022). Recently, Chun et al., demonstrated antibacterial activity against *Staphylococcus aureus* using surface-displayed expression of *S. aureus* phage endolysin LysSA11 on *S. cerevisiae* (Chun et al., 2020). In this research, we utilize recombinant yeast surface-expressed endolysin LysKB317 [a muramidase active domain with a SH3b-like cell wall binding domain; (Liu et al., 2015; Lu et al., 2020)] to combat bioethanol fermentation contamination of *L*. *fermentum* in a small-scale corn mash fermentation (Bischoff et al., 2007). Here, we show that surface-displayed LysKB317, in both N-terminus and C-terminus orientations anchored by mannoprotein, a-agglutinin [Aga2p; (Andreu and Del Olmo, 2013)], can remain active up to 72 h in the complex matrix of corn mash. This technology allows proactive treatment of contaminants, which can improve ethanol production by suppressing unwanted lactic and acetic acids through inhibition of target bacteria.

## Materials and methods

### Cultures and growth condition

All bacterial and yeast strains are described in Table 1, and unless otherwise stated, were grown in the following media: All *Escherichia coli* strains were grown in LB Miller (LB broth) culture medium (Difco Laboratories, Inc.); *L. fermentum* was grown in Lactobacilli MRS (MRS broth; Difco Laboratories, Inc.) media; and *S. cerevisiae* strains were grown in synthetic media with glucose (SDCAA; 2% D-glucose, 0.67% yeast nitrogen base (Cat# Y0626-250G; Sigma-Aldrich Inc.) with amino acid supplement minus tryptophan (-Trp; Cat# Y1876; Sigma-Aldrich Inc.), 0.5% peptone, 0.54% sodium phosphate dibasic anhydrous, and 0.86% sodium phosphate monobasic monohydrate; Sigma-Aldrich Inc.) without induction. To induce yeast constructs with the GAL1 galactose promoter, synthetic media SGCAA (-Trp) containing galactose (2% D-glucose was replaced with 2% galactose) were used to grow cells overnight with autotrophy selection. The control *S. cerevisiae* EBY100 strain was grown in SDCAA with complete amino acid supplementation. Antibiotics were added to LB media where appropriate at the following concentrations: carbenicillin (Car; Sigma-Aldrich Inc.) at 100 μg/mL or kanamycin (Kan; Sigma-Aldrich Inc.) at 50 μg/mL. *E. coli* cultures were grown at 37°C with agitation at 200 rpm and *L. fermentum* at 37°C stationary. *S. cerevisiae* strains were grown at 30°C with agitation at 200 rpm.

**TABLE 1:** Bacteria and yeast strains used in this experiment.

Bacteria and yeast	Relevant genotype/phenotype^a^ ^,^ ^b^ ^,^ ^c^	Reference or source
*Escherichia coli*		
Top10	F-*mcrA* Δ(*mrr-hsd*RMS-*mcr*BC) Φ80*lac*ZΔM15 Δ *lac*X74 *rec*A1 *ara*D139 Δ(*araleu*)7697 *gal*U *gal*K *rps*L (StrR) *end*A1 *nup*G	Invitrogen
Top10/pYD1	^a^Amp^R^, containing pYD1 plasmid	
Top10/pYD1::LysKB317	^a^Amp^R^, containing pYD1 plasmid and LysKB317 gene	This study
Top10/pYD5	^a^Amp^R^, containing pYD5 plasmid	
Top10/pYD5::LysKB317	^a^Amp^R^, containing pYD5 plasmid and LysKB317 gene	This study
Top10/pET21a::Aga2p-V5-LysKB317	^a^Amp^R^, containing Aga2p mature peptide V5 epitope tag (G_4_S)_3_ linker and LysKB317 gene	This study
E. cloni 10G/pRham N-His Kan::LysKB317	^b^Kan^R^, containing LysKB317 gene	Lu et al. (2020)
*Limosilactobacillus fermentum*		
0315-25	^c^Wildtype	Rich et al. (2015), Lu et al. (2020)
*Saccharomyces cerevisiae*		
EBY100	MATα AGA1::GAL1-AGA1::URA3 ura3-52 trp1 leu2-Δ200 his3-Δ200 pep4::HIS3 prbd1.6R can1 GAL	ATCC
EBY100/pYD1	Containing pYD1 plasmid	This study
EBY100/pYD1::LysKB317	Containing pYD1 and LysKB317 gene	This study
EBY100/pYD5	Containing pYD5 plasmid	This study
EBY100/pYD5::LysKB317	Containing pYD5 and LysKB317 gene	This study

^a^ Amp^R^, ampicillin-resistant.

^b^ Kan^R^, kanamycin-resistant.

^c^ Wildtype microbial strain was isolated from a Midwestern dry-grind fuel ethanol plant and selected from a previous screen (Rich et al., 2015).

### Constructs and plasmids

**FIGURE 1:**
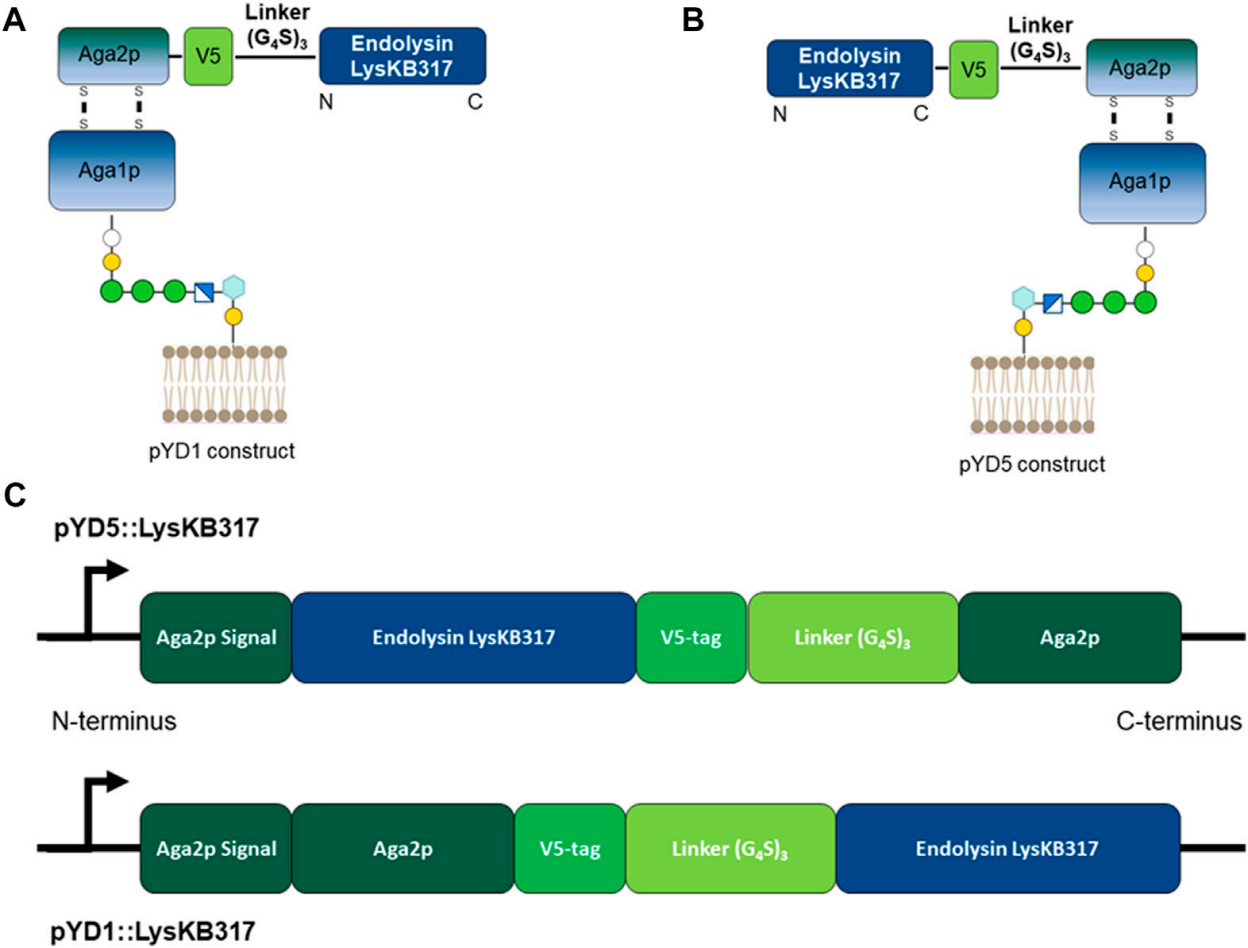
Schematic representation of *S. cerevisiae* Aga2p anchored displayed endolysin LysKB317 constructs. **(A)**. Amino-terminus glycine serine (G_4_S)_3_ linked LysKB317 in the pYD1 vector. **(B)**. Carboxyl-terminus glycine serine (G_4_S)_3_ V5 epitope tag linked LysKB317 in the pYD5 vector. **(C)**. Fusion endolysin LysKB317 nucleotide sequence insertion in the pYD1 and pYD5 vectors in relation to the Aga2p signal and mature Aga2p protein.

The endolysin LysKB317 (GenBank: KP027015.1) gene template from pRham N-His Kan::LysKB317 [Table 1; (Lu et al., 2020)] was PCR-amplified using primers described in Table 2. Amplified LysKB317 was inserted into the reversed PCR-linearized pYD5 vector [generously gifted by Dr. Mark Dumont (Wang et al., 2005)] using the Gibson cloning kit (New England BioLabs Inc.). To construct pYD5::LysKB317, the endolysin gene was inserted between the Aga2 signal cleavage site and upstream of V5–epitope and the glycine serine (G_4_S)_3_ linker (Figures 1A, C), exposing the N-terminus enzymatic domain of LysKB317 upon expression. To construct pYD1::LysKB317, synthetic genes containing a V5-epitope tag, a (G_4_S)_3_ linker, and the endolysin LysKB317 in the backbone of pET21a+ listed in Table 1 (GenScript) were used as DNA templates for PCR amplification using primers listed in Table 2. The vector pYD1 (Addgene plasmid # 73447) was linearized with primers (Table 2), and a PCR-amplified endolysin fragment was inserted between the end of Aga2p and stop codon after the 6xHis-tag, deleting the original (G_4_S)_3_ linker, enterokinase site, V5 tag, and His6 tag using Gibson cloning and exposing the C-terminus cell wall binding domain of LysKB317 upon expression. Constructs were transformed into *E. coli* TOP10 chemically competent cells (Invitrogen) and verified in-house by Sanger sequencing prior to *S. cerevisiae* EBY100 transformation using methods described by Gietz and Schiestl, (2007).

**TABLE 2:** Primers used to construct pYD1::LysKB317 and pYD5::LysKB317 endolysin.

Primer name	Primer sequence (5′-3′)	Purpose	References
pYD1KB317_VF	CAT​TCG​GAA​CCT​TTA​AAT​GAG​TTT​AAA​CCC​GCT​GAT​CTG​ATA​ACA	To amplify pYD1 vector for Gibson cloning	This work
pYD1KB317_VR	CCA​CCA​GAA​CCA​CCA​CCA​CCA​CTA​GCC​TGC​AG		
pYD1KB317_FF	ATG​TTT​TTA​AGC​TTC​TGC​AGG​CTA​GTG​GTG​GTG​GTG​GT	To amplify the endolysin LysKB317 gene for Gibson cloning	This work
pYD1KB317_FR	TCA​GAT​CAG​CGG​GTT​TAA​ACT​CAT​TTA​AAG​GT		
pYD1_F	TAA​TAC​GAC​TCA​CTA​TAG​GGA	To verify the vector construct	This work
pYD1_R	GGA​AAA​CAT​GTT​GTT​TAC​GG		
pYD5KB317_VF	GAA​TTC​GGT​AAG​CCT​ATC​CCT​AAC​CC	To amplify the pYD5 vector for Gibson cloning	This work
pYD5KB317_VR	TGC​TAA​AAC​GCT​AGC​AAT​AAC​AGA​AAA​TAT​TGA​AA		
pYD5KB317_FF	GCT​AGC​GTT​TTA​GCA​ATG​GCA​CTT​TAC​GTA​GTT​GAC​GTT	To amplify the endolysin LysKB317 gene for Gibson cloning	This work
pYD5KB317_FR	AGG​CTT​ACC​GAA​TTC​TTT​AAA​GGT​TCC​GAA​TGC​TTC​GCC		
pYD5_F	TAA​TAC​GAC​TCA​CTA​TAG​GGA​ATA​TTA​AGC	To verify the vector construct	This work
pYD5_R	AGT​GGG​AAC​AAA​GTC​GAT​TTT​G		

### Bacterial and yeast culture growth curves

Relative backscattering at the gain = 10 setting was read in a FlowerPlate 48-well microtiter plate with a clear flat bottom and aluminum sealing cover (Millipore Sigma) using a BioLector plate reader (Beckman Coulter). A triplicate of each strain at an OD_600 nm_ of 0.6 was seeded at a ratio of 1:100 (10 µL bacteria or yeast to 990 µL appropriate media) with a final volume of 1 mL measured for 72 h at 30°C and 400 rpm.

### Immunostaining and microscopy

A confocal microscope (Nikon Eclipse C1 Plus; Nikon) was used to image surface-displayed endolysin LysKB317 in *S. cerevisiae*. Induced or uninduced yeast cells were grown in appropriate SGCAA (induced) or SDCAA (uninduced) media at 30°C with 200 rpm overnight. Yeast cells (100 uL) were washed twice with 100 µL 1x phosphate-buffered saline (PBS; ThermoFisher) + 3% bovine serum albumin (BSA; ThermoFisher; as a stabilizing agent for immunostaining) buffer, pH 7.4 at 800 x *g* for 2 min to acclimatize yeast cells. Primary rabbit anti-LysKB317 (1:500; ThermoFisher) and secondary goat anti-rabbit Alexa 488 (1:5,000; Invitrogen) were incubated with the samples at room temperature for 30 min with gentle mixing. Cells were washed with 1x PBS + 3% BSA before visualizing a 5 µL resuspended yeast sample using a 488-nm argon laser with a 513/30 band pass emission filter on a photomultiplier tube (PMT) detector.

### Western blot analysis

Galactose-induced yeast cells were harvested and boiled in a water bath with 2x Laemmli sample buffer at a ratio of 1:1 for 10 min (Bio-Rad). Protein samples were separated on a sodium dodecyl sulfate (SDS)—polyacrylamide gel (Bio-Rad Any kD tris-glycine Mini-Protean TGX precast protein gel) as per the manufacturer’s instructions. Following SDS-PAGE, proteins were transferred onto a 0.2-µm pore size low-fluorescence polyvinylidene difluoride (PVDF) membrane (Bio-Rad) using a Trans-Blot Turbo transfer system (Bio-Rad). Transfer of protein was verified using Ponceau S Staining (Cell Signaling Technology, Inc.). Membrane non-specific blocking was performed with Blocker BSA in TBS (ThermoFisher) at room temperature for 1 h. Rabbit anti-LysKB317 antibody (1:500; ThermoFisher) was applied and incubated overnight with gentle swirling at 4°C in tris-buffered saline (TBS) containing 0.1% Tween-20 (Sigma-Aldrich Inc.). Secondary goat anti-rabbit antibody conjugated to Alexa 488 at a ratio of 1:5,000 (ThermoFisher) was applied and incubated with gentle swirling at room temperature. Membrane protein bands were detected using the ChemiDoc XRS+ imaging system (Bio-Rad).

### Live/dead viability assay

Induced/uninduced yeast cells of 500 µL at OD_600 nm_ = 2 and target bacteria of 500 µL at OD_600 nm_ = 2 were washed separately with ultrapure water twice at 800 x *g* for 2 min and dilute bacteria and yeast at 1:1,000 with ultrapure water. Live/dead stain (Invitrogen) was added immediately to the washed bacteria and incubated at room temperature for 10 min before mixing the bacteria to the yeast in a sterile fresh 1.5-mL Eppendorf tube. In triplicate biological repeats (*n* = 3), 5 µL of the yeast–bacteria sample was transferred into an SD025 chamber slide (Nexcelom), and the live and total bacteria were counted every 5 min with Cellometer X2 (Nexcelom) using a 0.5–5 μm cell size gating parameter.

### Small-scale corn fermentation

The fermentation method was adopted from Khatibi et al. with some modification (Bischoff et al., 2010; Khatibi et al., 2014). Briefly, corn mash (approximately 33% solids) was obtained from a commercial dry grind ethanol fermentation facility and stored at −20°C until use. In each 25-mL Erlenmeyer flask, 12.0 mL of autoclaved corn mash was pretreated with 10 µL glucoamylase (Allcoholase II Liquid 300; Alltech) and 200 µL 12% (NH_4_)_2_SO_4_ overnight at 37°C with agitation at 200 rpm. About 6.8 mL of 25% galactose for induction or ultrapure water (for non-induction) was added to the flasks to a final concentration of 20% solid corn mash and 10% galactose as described previously (Khatibi et al., 2011; Khatibi et al., 2014). Inoculation of yeast strains (induced/uninduced or untransformed), 1 mL of OD_600 nm_ = 160 and bacteria, 200 µL of OD_600 nm_ = 8 (or ultrapure water for control flask without infection), was added for a final volume of 20 mL per fermentation flask. Rubber stoppers with a G20 1½ PrecisionGlide Needle (Becton Dickinson) to vent gas (CO_2_) were used as closures. Fermentation flasks were incubated at 30°C with 50 rpm for 72 h. At time points 0 h, 24 h, 48 h, and 72 h, 500 µL of the sample was removed for analysis, and the flasks were immediately returned to the incubator. Samples at each time point were diluted at a ratio of 1:100,000 in fresh sterile 1.5-mL Eppendorf tubes with ultrapure water, plated using an Eddy Jet 2 spiral plater (E mode 50; IUL Instruments) on MRS-agar containing 10 μg/mL cycloheximide (Sigma-Aldrich, Inc.), and incubated at 37°C overnight. Colonies were counted using a Flash & Go plate reader (IUL Instruments) to determine the CFU/mL, where ten colonies or more were counted. The remaining 500-µL samples were spun-down using a benchtop centrifuge at maximum speed for 1 min, and 200 µL of the supernatant was removed for HPLC analysis.

### HPLC metabolite analysis

A high-performance liquid chromatography (HPLC) system (Shimadzu) with a 300-mm Aminex HPX-87H column (0.5 mL/min, 5 mM H_2_SO_4_ at 65°C; Bio-Rad) and a refractive index detector (RID) was used to detect fermentation metabolites [acetic acid, ethanol, glucose, and lactic acid (Bischoff et al., 2010)].

### Expression and purification of His-tagged LysKB317

A single colony of *E. coli* harboring pRham N-His Kan::LysKB317 (Table 1) was isolated and inoculated into 5 mL LB media with Kan at 37°C and 200 rpm shaking overnight. This was used to inoculate fresh 25 mL LB with Kan at a ratio of 1:100, which was then incubated at 37°C and 200 rpm until the OD_600 nm_ reached 0.6 (Lu et al., 2020). Culture was induced with 0.2% (w/v) L-rhamnose (Sigma-Aldrich Inc.) for at least 16–18 h. Cells were centrifuged at 8,000 x *g* for 15 min at 4°C, and the spent medium was decanted. Cell pellets were then lysed using B-Per (Invitrogen) with DNase I, RNase I, and lysozyme (10 U/mL; ThermoFisher) and incubated at room temperature for 20 min. The whole cell lysate was centrifuged at 15,000 x *g* for 5 min and the soluble supernatant was pipetted into a His-Spin column (ZymoResearch) following the manufacturer’s protocol for Ni-NTA protein purification. The concentration of purified protein was quantified using the Qubit Protein Assay Kit (ThermoFisher) and subsequently used as a positive control for SDS-PAGE and Western blot analysis.

### pH measurement of the spent synthetic SDCAA media

Spent medium approximately 500 µL was pipetted into a 1.5-mL Eppendorf tube in triplicate. A micro-pH electrode (Hl1093B, Hanna Instruments) was inserted into the medium to measure the pH.

### Statistical analysis

Where appropriate, experimental results were analyzed using two-way analysis of variance (ANOVA) with the Tukey post-hoc test to determine statistical significance at *p < 0.05 (GraphPad Prism version 9.5.1).

## Results

Fluorescence microscopy examined the *S. cerevisiae* surface-displayed endolysin LysKB317

**FIGURE 2:**
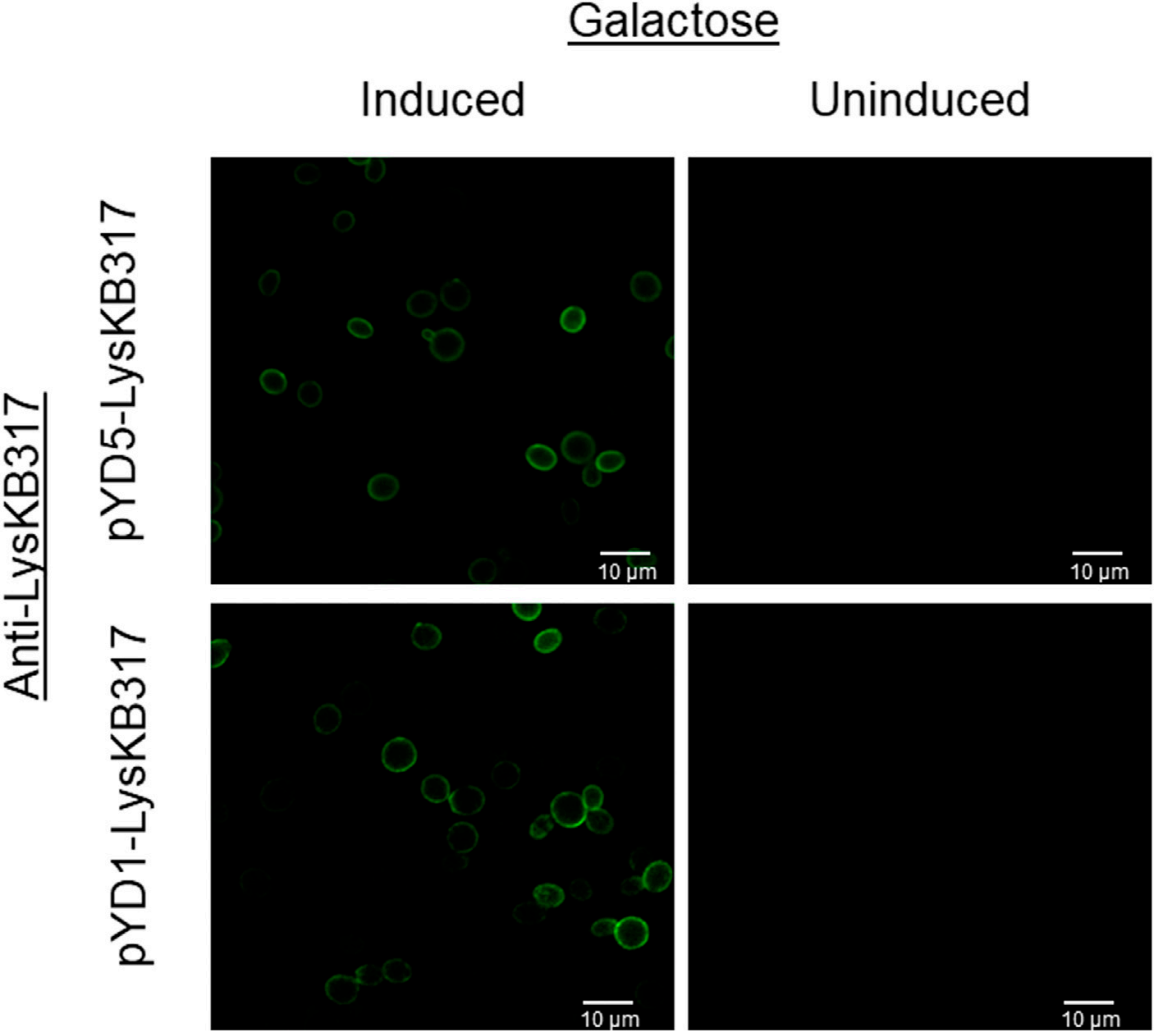
Fluorescent microscopy imaging of surface yeast-displayed LysKB317. Immunostaining utilizing anti-LysKB317 DyLight488 conjugated antibody to detect galactose-induced pYD1 and pYD5 constructs. Non-induced (glucose media) transformed *S. cerevisiae* EBY100 did not result in a detectable fluorescent signal from the immunostaining.

We constructed endolysin LysKB317 expression vectors (pYD1 and pYD5) with gene inserts N-terminus linked (pYD1::LysKB317; Figure 1A) and C-terminus linked (pYD5::LysKB317; Figure 1B). The constructs expressed in *S. cerevisiae* strain EBY100 (Figure 1C) were confirmed by Western blot analysis (Supplementary Figure S1). Upon verification of endolysin expression, LysKB317 immunostaining was performed on whole induced yeast cells. Cells with empty vectors pYD1 and pYD5 did not yield a positive signal for LysKB317 (results not shown). Yeast constructs harboring both pYD1 and pYD5 endolysin constructs subjected to galactose induction resulted in a positive fluorescent signal against LysKB317 *via* Alexa488 (Figure 2). Fluorescence was localized to the extracellular interface of the cells. No detectable signal was found for LysKB317 on the surface of yeast when cells were grown under non-induced conditions (SDCAA -Trp).

### Surface-expressed endolysin LysKB317 lysis of target bacteria

**FIGURE 3:**
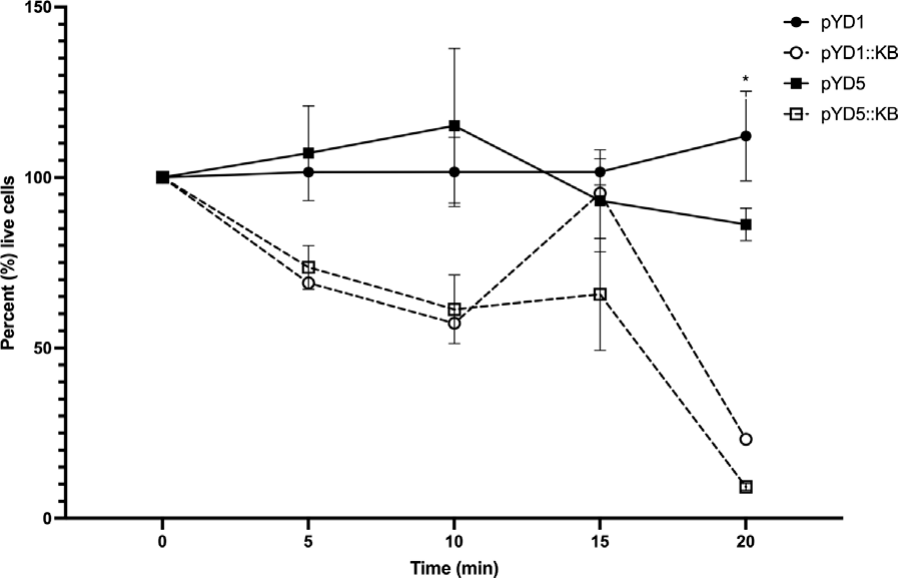
Measurement of total percent of live *L. fermentum* 0315-25 cells over 0, 5-, 10-, 15-, and 20-min treatments after exposure to galactose-induced *S. cerevisiae* EBY100/pYD1 (pYD1; black line black circle), pYD1::LysKB317 (pY1::KB; black line white circle), pYD5 (pYD5; gray line black circle), and pYD5::LysKB317 (pKD5::KB; gray line white circle). Result measurements were expressed in percent (%) live cells (*n* = 3 independent replicates, and error bar = SEM). *p < 0.05 compared to pYD1 plasmid control and pYD5 plasmid control against pYD1::KB and pYD5::KB. No significant difference found at 20 min between pYD1::KB and pYD5::KB.

To measure the lytic activity of yeast surface-expressed endolysin against *L. fermentum,* a live/dead viability assay was conducted with all four constructs (pYD1, pYD1::LysKB317, pYD5, and pYD5::LysKB317; Table 1). The total live bacterial cell count was measured every 5 minutes after mixing the yeast and bacteria (Figure 3). All yeast constructs harboring an empty plasmid (pYD1 or pYD5) showed a 9.5% decrease in live bacteria cells compared to an 83.8% decrease in live cells in constructs expressing the LysKB317 endolysin (pYD1::LysKB317 or pYD5::LysKB317) over a 20-min incubation time frame at room temperature. Endolysin-expressing constructs demonstrated most inhibitory activity against target bacteria at the 20-min time point. No significant differences in inhibitory effect between endolysin-expressing constructs were seen between pYD1::LysKB317 and pYD5::LysKB317 (*p* = 0.59). At the end of 20 min, endolysin-expressed constructs were at approximately 9.25%–23.19% live bacterial cells *L. fermentum* left compared to empty plasmid controls (pYD1 and pYD5) with 84%–97% live cells. There was no significant difference between the pYD1 and pYD5 plasmid control samples (*p* = 0.42). Statistically significant differences (*p* < 0.05) were seen at 20 min by comparing pYD1 versus pYD1::KB (*p* = 0.023) and pYD5 versus pYD5::KB (*p* = 0.007).

### Viable bacteria decreased over 72 h in corn mash fermentation

**FIGURE 4:**
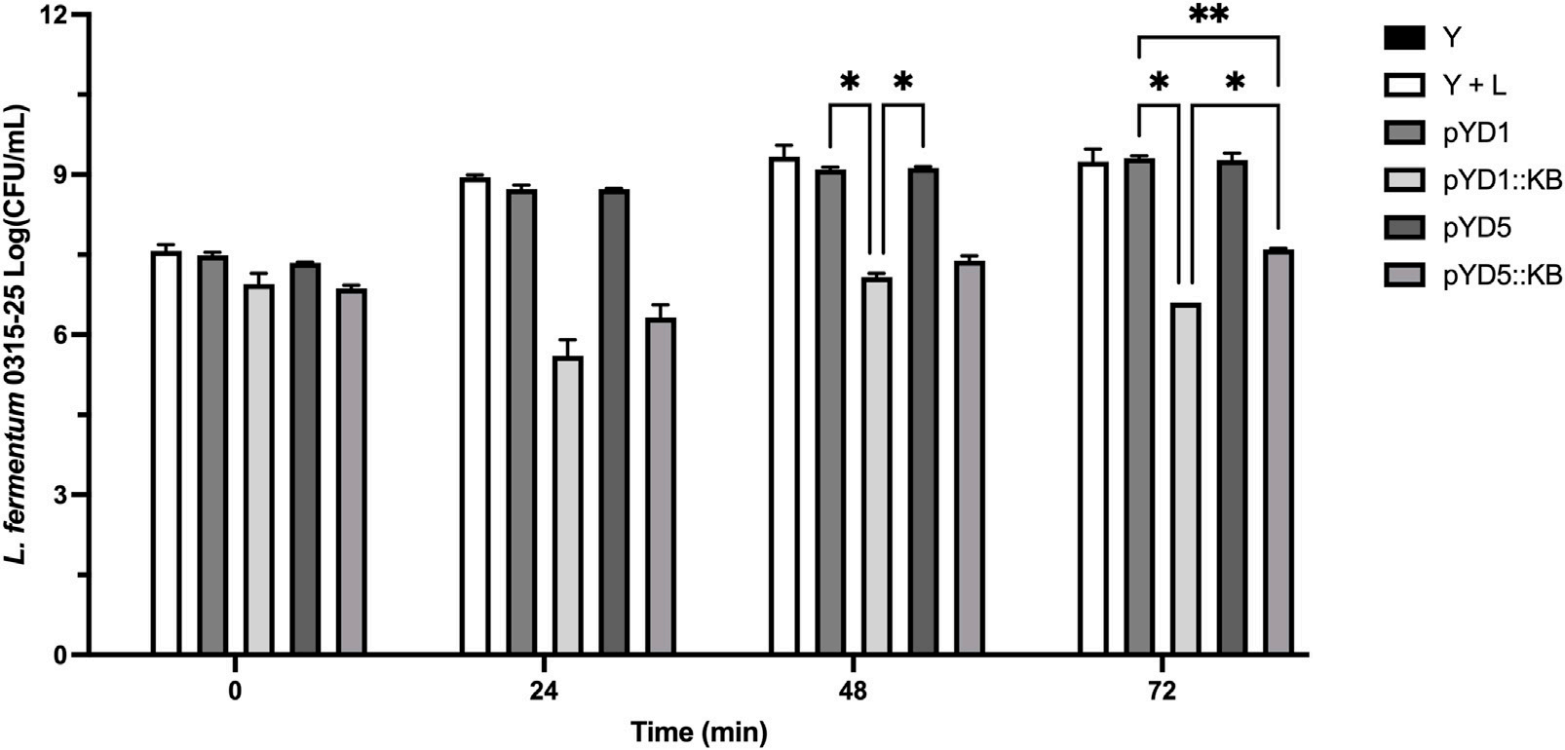
Small-scale corn mash fermentation. Galactose-induced *S. cerevisiae* EBY100 constructs were challenged with *L. fermentum* 0315-25 for a period of 72 h: yeast-only control, no bacterial challenge (Y; black bar), yeast with bacteria (Y + L; white bar), yeast with empty vector pYD1 plus bacteria (pYD1; dim gray bar), yeast with induced endolysin LysKB317 in pYD1 vector plus bacteria (pYD1::KB; silver gray bar), yeast with pYD5 empty vector plus bacteria (pYD5; gray bar), and yeast with induced endolysin LysKB317 in pYD5 vector plus bacteria (pYD5::KB; light gray bar). The spiral plate technique was performed, and log CFU/mL was measured to determine cell counts at time-points 0, 24, 48, and 72 h. Result measurements were expressed in log CFU/mL (*n* = 2 independent replicates and error bar = SEM). *p < 0.05 compared to yeast infection control based on two-way analysis of variance (ANOVA).

In the negative control, yeast-only sample, no measurable bacterial level (> 3-log CFU/mL) was detected from 0 h–72 h (Figure 4). Yeast samples with positive controls challenged with *L. fermentum* infection showed a steady log CFU/mL increase over a period of 48 h from 7.6–9.4 log CFU/mL and a slight decrease to 9.3 log CFU/mL at 72 h. Yeast constructs with empty control vectors (pYD1 and pYD5) challenged with bacterial infection over a period of 72 h showed an overall increase in bacterial load of 7.5–9.3 log CFU/mL and 7.3–9.3 log CFU/mL, respectively. In comparison, yeast-carrying constructs expressing the endolysin LysKB317 (pYD1::LysKB317 and pYD5::LysKB317) challenged with bacterial infection significantly lowered the log CFU/mL count from 7.0–6.6 log CFU/mL (*p* = 0.021) and 6.9–7.6 log CFU/mL (*p* = 0.022), respectively, at the end of 72 h. Overall, at least 2.7 log CFU/mL and 1.7 log CFU/mL log reduction was observed in pYD1 and pYD5 endolysin constructs compared to yeast infection challenge control alone. Statistical significance at 72 h was detected between yeast (Y) and infection control (Y + L; *p* = 0.023). No significance was detected when comparing infection control (Y + L) with plasmid controls (pYD1 or pYD5).

### Fermentation metabolite analysis with HPLC

**FIGURE 5:**
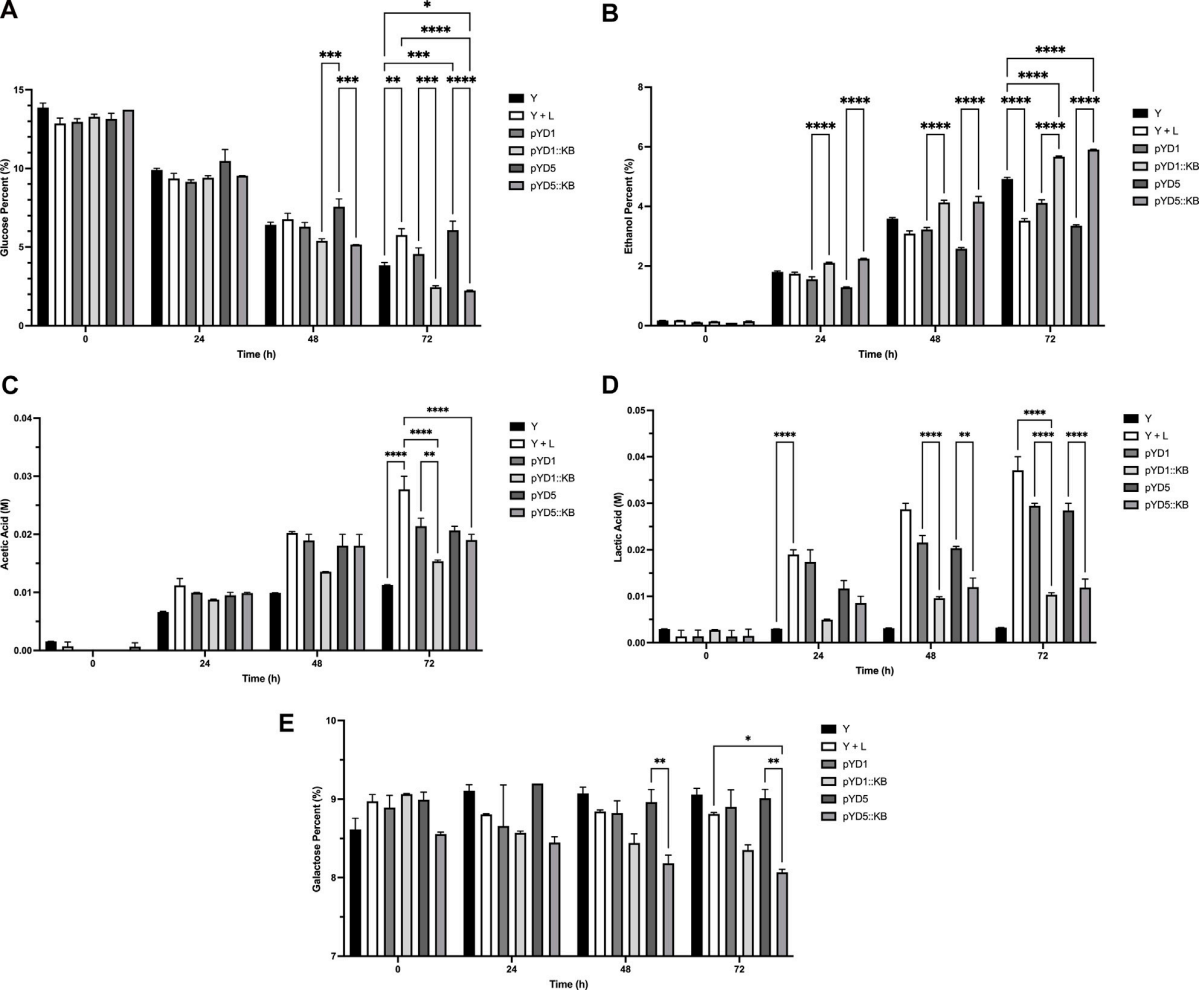
HPLC analysis of fermentation metabolites over 72 h. All concentration of ethanol, glucose, and galactose are defined as [g/100 mL] and are referred to as percent (%). Acetic and lactic acids are defined as molar (M) concentrations. **(A)** Percent glucose (%), **(B)** percent ethanol (%), **(C)** acetic acid (M), **(D)** lactic acid (M), and **(E)** percent galactose (%) utilizations were performed in duplicates and measured at 0, 24, 48, and 72 h compared between treatments. Yeast-only control (Y; black bar), yeast with bacteria (Y + L; white bar), yeast with empty vector pYD1 plus bacteria (pYD1; dim gray bar), yeast with induced endolysin LysKB317 in pYD1 vector plus bacteria (pYD1::KB; silver gray bar), yeast with pYD5 empty vector plus bacteria (pYD5; gray bar), and yeast with induced endolysin LysKB317 in pYD5 vector plus bacteria (pYD5::KB; light gray bar). Treatments (*n* = 2; error bar = SEM) with *p < 0.05 for statistical significance using two-way analysis of variance (ANOVA).

The average glucose percent level at the beginning (0 hour) for the corn mash fermentation flasks was at 13.31% (Figure 5A). The level of glucose after 72 h of fermentation decreased from 13.31% to 3.85% for the *S. cerevisiae-*only sample, compared to 5.75% in yeast with bacterial infection. Plasmid control samples (pYD1 and pYD5) at the end of 72 h showed a glucose level ranging from 4.56%–6.07%, with an average glucose percent level of 5.31% (Figure 5A Table 3). Yeast strains expressing LysKB317 had percent glucose levels of 2.45% (pYD1::LysKB317) and 2.24% (pYD5::LysKB317) after 72 h of fermentation, which are significantly lower than those of the bacterial infection control samples (*p* < 0.0001). Statistically significant differences were observed between yeast control (Y) and infection control (Y + L) with *p* < 0.002). At 72 h, glucose utilization between plasmid controls (pYD1 or pYD5) with LysKB317 constructs (pYD1::KB or pYD5::KB) showed statistical significance at the *p*-value lower than 0.0008. No significant differences were detected between the infected control (Y + L) with pYD1 (*p* = 0.1) and pYD5 (*p* = 0.9). The average percent ethanol generated across all samples was 0.14% at time 0 (Figure 5B). At the end of 72 h of fermentation, yeast-only control samples generated 4.92% ethanol, which is significantly higher than that of infection control, which is 3.52%. Yeast with plasmid control samples showed similar levels of percent ethanol ranging from 3.35% to 4.11% (pYD1 and pYD5; Table 3). Significant increases were observed in endolysin-expressed constructs (5.67%–5.91% ethanol) compared to the infection control samples, which had 3.52% ethanol (*p* < 0.0001). Accumulation of acetic acid at the end of 72-h fermentation in yeast with infection (0.026 M) was significantly higher than that of the yeast-only control (0.011 M; *p* < 0.0001) and plasmid controls (0.022 M and 0.023 M; *p* < 0.0001) for pYD1 and pYD5. The acetic acid concentration is also significantly lower for endolysin-expressed constructs (0.015 M and 0.018 M for pYD1::LysKB and pYD5::LysKB; *p* < 0.0001) than for the infection control (0.026 M). No significance in acetic acid level at 72 h was detected between pYD1::KB and pYD5::KB (Figure 5C). The level of lactic acid accumulation in the no-infection control was 0.003 M (Figure 5D), while the bacterial infection samples had 0.035 M lactic acid at the end of 72 h, a 984% increase compared to that of no infection control. Plasmid control construct samples resulted in 0.028 M lactic acid for both pYD1 and pYD5, an 18.1% and 19.3% respective decrease in lactic acid compared to infection control. Endolysin-expressed pYD1::KB and pYD5::KB resulted in 0.010 M (70.3% reduction; *p* < 0.0001) and 0.014 M (60.2% reduction; *p* < 0.0001) in lactic acid, respectively, compared to infection fermentation control (Table 3). Due to galactose utilization genes being repressed in the presence of glucose in the fermentation flasks, the level of galactose at the initial time of 0 h averaged at 8.85% and remained practically the same at the end of 72 h of fermentation, averaging at 8.70% (Figure 5E). There is no significant difference in galactose utilization between yeast and infection control, pYD1 vs. pYD1::KB and pYD5 vs. pYD5::KB.

**TABLE 3:** Small-scale corn mash fermentation HPLC metabolite analysis and Log CFU/mL plating at 72 h.

Treatment	*L. fermentum* 0315–25	Fermentation metabolites			
	Log (CFU/mL)	Ethanol (%)	Glucose (%)	Lactic acid (M)	Acetic acid (M)
EBY100	< 3.0	4.9 ± 0.1	3.8 ± 0.2	0.0032 ± 0.0000	0.0113 ± 0.0000
EBY100 + *L. fermentum*	9.2 ± 0.24	3.5 ± 0.1	5.8 ± 0.4	0.0347 ± 0.0005	0.0255 ± 0.0000
EBY100/pYD1 + *L. fermentum*	9.3 ± 0.04	4.1 ± 0.1	4.6 ± 0.4	0.0284 ± 0.0006	0.0226 ± 0.0002
EBY100/pYD1::LysKB317 + *L. fermentum*	6.6 ± 0.01	5.7 ± 0.0	2.4 ± 0.1	0.0103 ± 0.0005	0.0154 ± 0.0002
EBY100/pYD5 + *L. fermentum*	9.3 ± 0.13	3.4 ± 0.0	6.1 ± 0.6	0.0280 ± 0.0010	0.0219 ± 0.0005
EBY100/pYD5::LysKB317 + *L. fermentum*	7.6 ± 0.02	5.9 ± 0.0	2.2 ± 0.0	0.0138 ± 0.0001	0.0181 ± 0.0000

## Discussion

Previously described endolysin LysKB317 (protein accession number AIY32273.1) is a 33-kDa peptidoglycan hydrolase with a glycoside hydrolase family (GH25) enzymatic domain and a SH3b cell wall binding domain that demonstrated lytic activity against most *Lactobacillus* and *Limosilactobacillus* species and some lactic acid bacteria (Lu et al., 2020). Yeast cell surface display technology improves the catalysis, stability, and activity of proteins and enzymes (Shiraga et al., 2005; Chen et al., 2011; Li et al., 2014). The effective use of cell surface display technology was demonstrated through bioprocessing of hemicellulose enzymes displayed on the *S. cerevisiae* cell surface to process corn cob for ethanol fermentation (Cunha et al., 2020). Yeast expression of endolysin has recently been used to express endolysin LysA, LysA2 (Khatibi et al., 2014; Kim et al., 2018). More recently, the yeast surface display of a staphylococcal phage endolysin LysSA11 has been used as an efficient platform for enzybiotic production to kill *S. aureus* (Chun et al., 2020). As a proof of concept, using such a strategy, we demonstrated that yeast surface-displayed lactobacilli phage endolysin such as the LysKB317 can proactively control bacterial contamination in commercial fuel ethanol fermentation facilities.

### Endolysin LysKB317 demonstrated bacterial lysis regardless of the linkage orientation of the enzyme

The use of Aga1—Aga2 anchor proteins (Boder and Wittrup, 1997) and a glycine serine (G_4_S)_3_ linker for yeast surface display allows a fusion protein to be expressed either on the N-terminus [pYD1; (Kieke et al., 1997)] or the C-terminus [pYD5; (Wang et al., 2005)] orientation to examine the efficacy of LysKB317 [Figure 1A; (Boder and Wittrup, 1997; Bertrand et al., 2016)]. Computational structures of the Aga2p-linked endolysin were predicted for both orientations [Supplementary Figure S2; (Baek et al., 2021)]. Both constructs were able to display on the yeast surface and observed through fluorescent microscopy with similar fluorescent intensities (Figure 2). Growth of yeast constructs in induced media (SGCAA; -Trp) and in uninduced media (SDCAA; -Trp) had no significant differences in growth profiles (Supplementary Figure S3). This was expected due to the yeast’s acclimation to glucose media prior to induction; its preference of glucose as the carbon source, compared to galactose; and the expression of recombinant fusion proteins (Ozcan, 2002; Demir and Kurnaz, 2006). Regardless of slower initial growth upon induction, yeast constructs carrying LysKB317 with N- or C-terminus linkage (pYD1::LysKB317 or pYD5::LysKB317) demonstrated significant lytic activity against target bacteria within the first 5 min of co-incubation, with only 9.3%–23.2% live bacteria cells remaining at the end of the 20-min incubation period in comparison to the plasmid control, which had between 86.2% and 112.2% live bacterial cells; Figure 3. No significant differences were detected between the plasmid control (pYD1 vs. pYD5), although the percent of live cells in pYD1 increased slightly by the 20-min time point. The speed at which the yeast surface-displayed LysKB317 started to lyse target bacteria within the first 5 min and accelerated by 20 min after co-incubation suggests the effectiveness of the system *in vitro*.

### Yeast surface-displayed endolysin LysKB317 can actively inhibit *L. fermentum* up to 72 h

Suppression of bacterial growth in a small-scale corn mash fermentation model (Bischoff et al., 2010; Roach et al., 2013; Khatibi et al., 2014; Lu et al., 2020) was evident in yeast-displaying endolysin (Figure 4). Over 72 h of corn mash fermentation, bacterial load started with an average of 7.3-log CFU/mL and completed with 6.6-log CFU/mL using pYD1::KB and 7.6-log CFU/mL for pYD5::KB (Table 3). In comparison, infection control and vector control samples showed continuous growth of bacteria in corn mash from an average of 7.3-log to 9.3-log CFU/mL in 3 days (Figure 4). When comparing the lytic activity of yeast surface-displayed endolysin LysKB317 in corn mash fermentation to that *in vitro* (Figure 3) and to the exogenous addition of 1 µM purified LysKB317 shown in our previous study [up to 4-log fold CFU/mL reduction (Lu et al., 2020)], the level of bacterial lysis over 72 h was not as prominent. Complex matrixes contained in corn mash fermentation can impede endolysin LysKB317 from contacting target *L. fermentum*. This is especially challenging given that surface-displayed endolysin is immobilized to yeast *via* glycine serine (G_4_S)_3_ linker compared to the exogenous addition of unbound endolysin and is limited to the finite amount of surface-displayed endolysin that was available in the pre-induced galactose media. Nonetheless, yeast constructs displaying LysKB317 reduced target bacteria by at least 1-log fold up to 2.7-log fold (Figure 4). Furthermore, optimization of the system by increasing anchor-linker length, replacing the galactose promoter (Gal1) with non-glucose-repressed promoters, plasmid integration, or replacing laboratory *S. cerevisiae* yeast with commercial strains could remove some of the limitations of the current system and result in a higher continuous expression output of endolysin at a faster yeast growth rate to outcompete the growth of *L. fermentum*.

### Metabolite analysis demonstrates active bio-mitigation against a bacterial contaminant in small-scale corn mash fermentation

At 72 h in corn mash fermentation, the yeast surface-displayed LysKB317 endolysin was successful in controlling *L. fermentum*, able to restore ethanol production (Table 3), and able to increase glucose utilization compared to infection and plasmid control (Figures 5A, B). It is important to note that laboratory *S. cerevisiae* strains such as EBY100 are considered less efficient at fermentation and growth compared to commercial *S. cerevisiae* ethanol-producing strains (Lin et al., 2012; Jansen et al., 2017). As previously mentioned, moving the yeast display system to a commercial yeast strain can improve yeast growth and glucose utilization to complete fermentation. It is worth noting that glucose consumption in yeast strains expressing the lysin improved compared to the yeast-only sample, which is speculated to have a higher energy requirement for yeasts expressing lysin (Figures 5A, B). Furthermore, replacing the galactose promoter and the use of a costly galactose carbon source with a glucose-inducible or -constitutive promoter would significantly improve our system. Given the competitive nature of glucose over galactose utilization by the yeast, minimal usage of galactose for the entire duration (72 h) of corn mash fermentation was observed (Figure 5E). The difference between pYD5 and pYD5::KB at 48 and at 72 h could be attributed to sampling variance.

### Stuck fermentation is prevented in corn mash fermentation by yeast surface-displayed LysKB317

In addition to lowering the pH, accumulation of acetic acid and lactic acid produced by LAB such as *L. fermentum* causes inhibition of sugar utilization and growth in *S. cerevisiae* to the point where the fermentation is stalled, also known as “stuck fermentation” (Salmon, 1989; Bisson, 1999; Bischoff et al., 2007; Liu et al., 2015). This stuck fermentation can happen in the *S. cerevisiae* EBY100 strain when the level of lactic acid reaches 0.03 M or higher (Figures 5A–D). We first examined the pH level of spent media (SDCAA) after culturing *S. cerevisiae* and yeast with bacteria (infection control) for 72 h. Yeast-only samples and yeast with infection both showed a decrease in pH over time; however, samples containing bacteria showed a consistent significant lower pH than that of yeast-only samples from 24 to 72 h (*p* < 0.0001; Supplementary Figure S4). The pH level averaged pH 3.4 which based on our previous report on LysKB317 may be below the endolysin’s optimal pH activity range of pH 5.5 (Lu et al., 2020). Nevertheless, yeast-displayed endolysin LysKB317 suppressed the level of lactic and acetic acids generated by the infection in corn mash below levels found in infection and plasmid control samples (Figures 5C, D) and prevented stuck fermentation (Table 3) by re-establishing healthy fermentation characteristics through limiting the growth of *L. fermentum* in the corn mash. *S. cerevisiae* surface display of endolysin LysKB317 demonstrated that this system can inhibit *L. fermentum* by at least one-log fold in small-scale corn mash fermentation. As a proof of concept, the yeast surface display platform could significantly express recombinant endolysins and demonstrated practicality by proactively mitigating LAB fermentation contaminants. This endolysin system, when optimized, is an ideal alternative to supplemental antibiotic treatment in biofuel fermentation facilities.

## Data Availability

The original contributions presented in the study are included in the article/Supplementary Material; further inquiries can be directed to the corresponding author.

## Author disclaimer

Mention of trade names or commercial products in this publication is solely for the purpose of providing specific information and does not imply recommendation or endorsement by the U.S. Department of Agriculture (USDA). The USDA prohibits discrimination in all its programs and activities on the basis of race, color, national origin, age, disability, and where applicable, sex, marital status, family status, parental status, religion, sexual orientation, genetic information, political beliefs, or because all or part of an individual’s income is derived from any public assistance program. The USDA is an equal opportunity provider and employer.
