# Supplementary material for: Saccharomyces cerevisiae surface display of endolysin LysKB317 for control of bacterial contamination in corn ethanol fermentations
Source: Front Bioeng Biotechnol. 2023 Apr 6;11:1162720. doi: 10.3389/fbioe.2023.1162720 (PMC10117863; doi:10.3389/fbioe.2023.1162720)
Supplement: Supplementary file 1 [file DataSheet1.PDF]

Supplemental materials

Saccharomyces cerevisiae surface display endolysin LysKB317 and application in corn ethanol fermentation bacterial control

Shao-Yeh Lu[1*], Siqing, Liu[1], Maulik Patel[2], Kristina M. Glenzinski[1] and Christopher D. Skory[1]

[1]USDA, Agricultural Research Service, National Center for Agricultural Utilization Research, Renewable Product Technology Research Unit, Peoria, IL 61604, USA

[2]Oak Ridge Institute for Science and Education (ORISE), United States

*Corresponding author:

Shao-Yeh Lu, Ph.D., USDA-ARS-NCAUR, 1815 N. University St., Peoria, IL 61604-3902, USA; (309) 681-6067; shao.lu@usda.gov

Figure S1. ***S. cerevisiae*** **surface displayed LysKB317.** Endolysin LysKB317 was induced with galactose in SG-CAA (-Trp) media overnight at 30°C with agitation. Western blot was performed by transferring protein samples onto polyvinylidene difluoride (PVDF) membrane and probed with anti-LysKB317 antibody detecting the presence of LysKB317. Endolysin LysKB317 can be detected in two forms. Aga2p-LysKB317 linked (estimated without glycosylation ~45 kDa) and purified LysKB317 (estimated without glycosylation ~33.8 kDa). PGK-1 is the housekeeping gene (~46 kDa) as loading control.

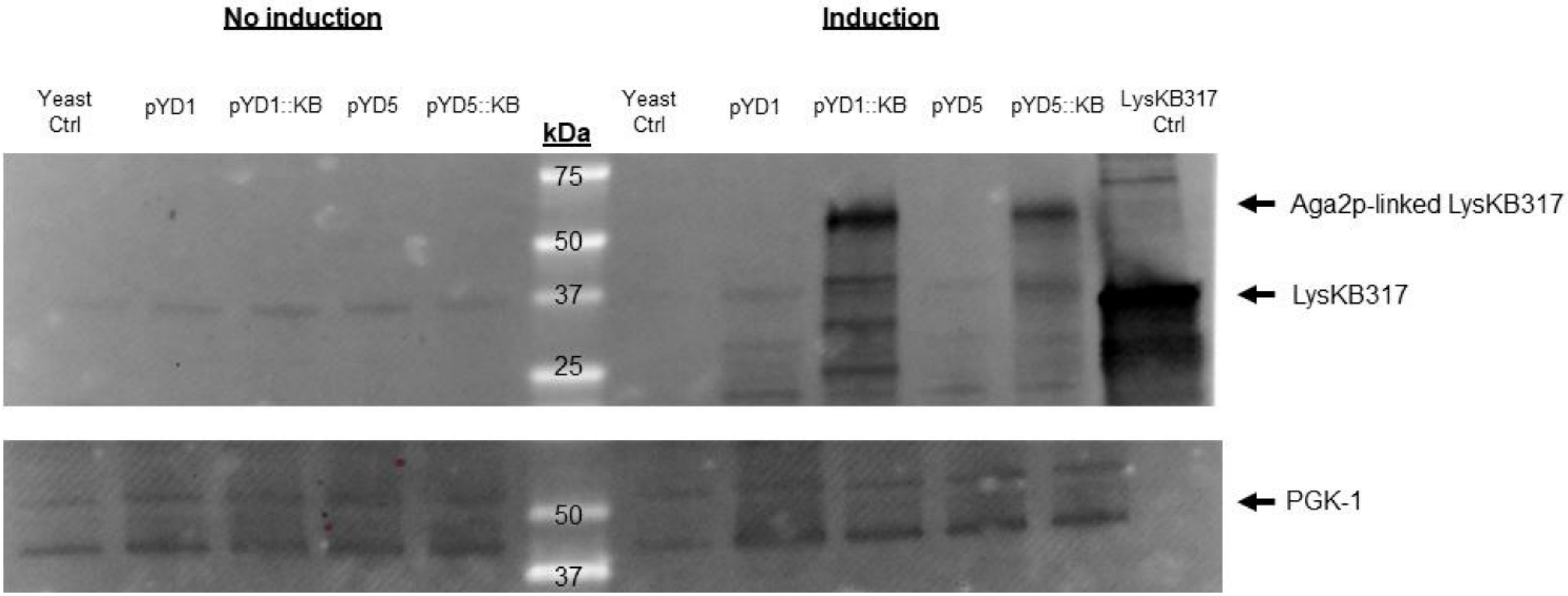

Figure S2. Tertiary structure prediction of LysKB317 linked to Aga2p. RoseTTAFold was used for protein structure predictions to N- or C-terminally linked endolysin LysKB317 (1). Enzymatic Active Domain (EAD), Cell wall Binding Domain (CBD), and the cell surface adhesion protein (Aga2p). (A) pYD1::LysKB317 construct and (B) pYD5::LysKB317.

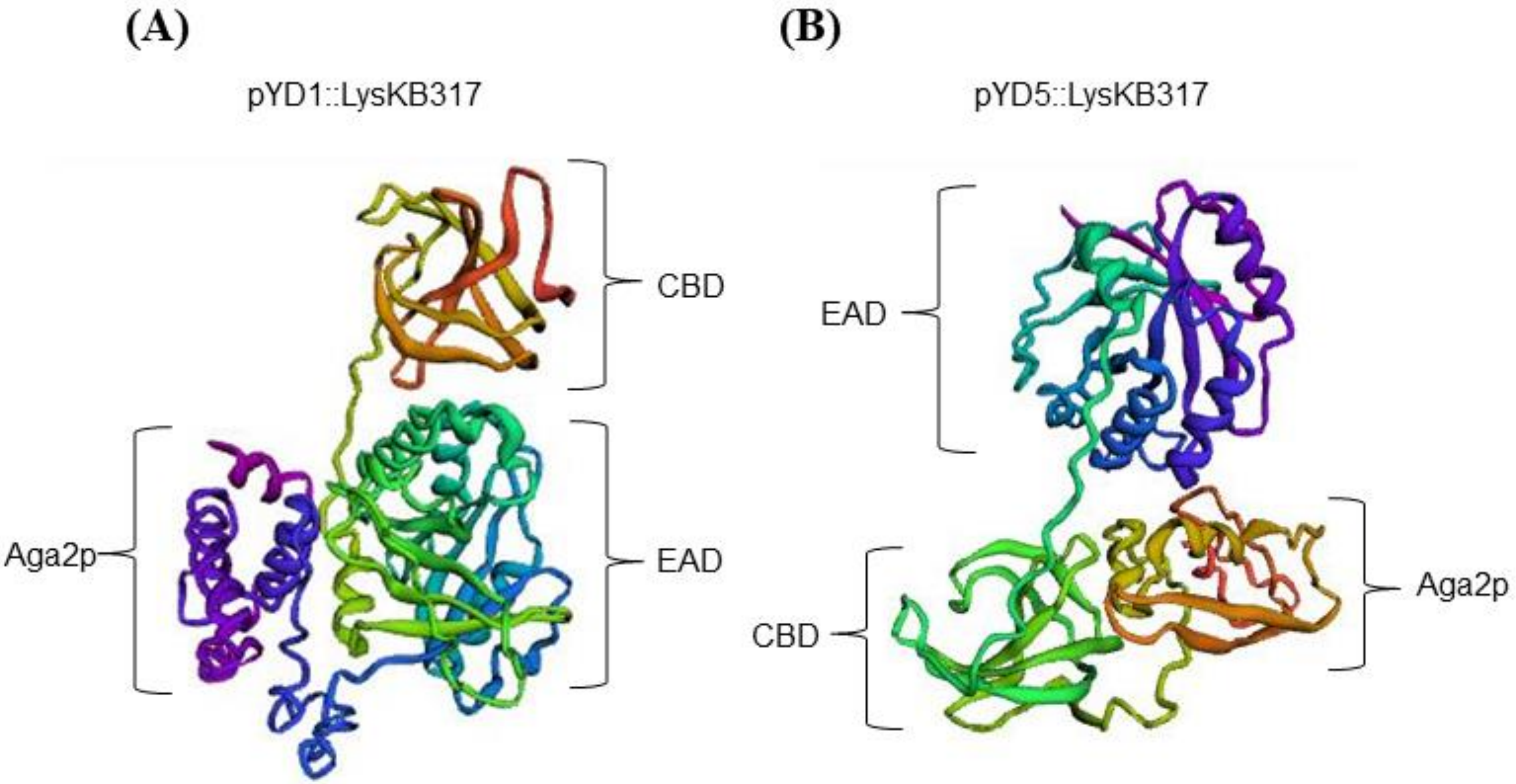

Figure S3. Relative backscatter signal of induced and uninduced *S. cerevisiae* EBY100 constructs. Yeast constructs without induction were grown in SD-CAA (-Trp) dropout media containing dextrose (D). Induced yeast and constructs were grown in SG-CAA (-Trp) dropout media containing galactose (G). Yeast strains (**Table 1**): EBY100/pYD1 (pYD1), EBY100/pYD1::LysKB317 (pYD1-KB), EBY100/pYD5 (pYD5), EBY100/pYD5::LysKB317 (pYD5-KB) were grown at 30°C for approximately 7 days. All measurements were done in triplicates.

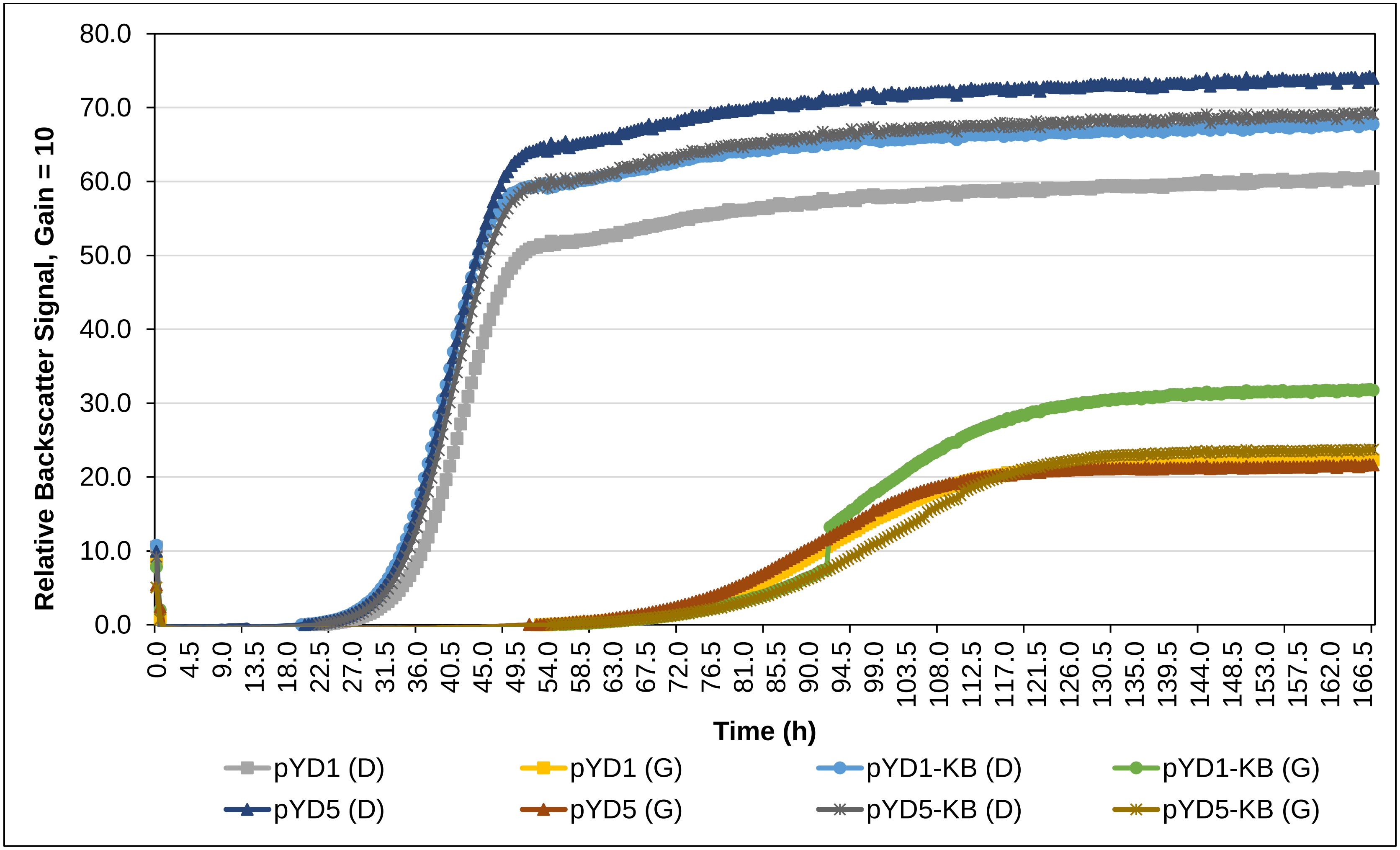

Figure S4. Spent media pH measurement of *S. cerevisiae* and co-culture infection with *L. fermentum*. The pH of the synthetic media SDCAA was measured in yeast only (Y; black bar) and yeast plus bacteria, *L. fermentum* 0315-25 (Y + L; white bar) for a period of 72 hrs. The pH containing bacterial infection (white) demonstrated a lower pH compared to the yeast only control after 24 hrs and remained lower till 72 hrs. All measurements were done in duplicates and statistically analyzed using two-way analysis of variance (ANOVA; ****$p < 0.0001$).

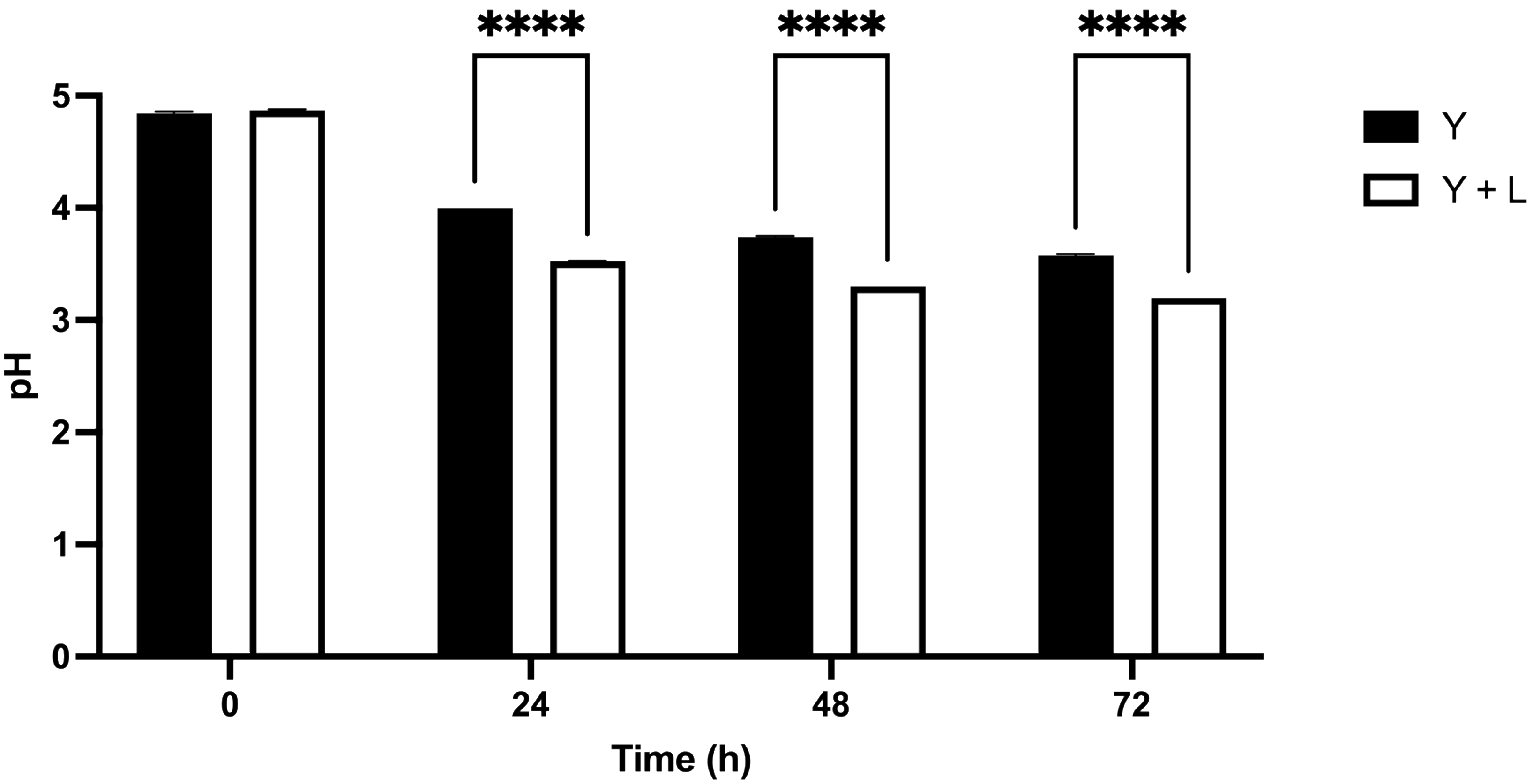

REFERENCE

Baek M, DiMaio F, Anishchenko I, Dauparas J, Ovchinnikov S, Lee GR, Wang J, Cong Q, Kinch LN, Schaeffer RD *et al*: Accurate prediction of protein structures and interactions using a three-track neural network. *Science* 2021, 373(6557):871-+.
